# Metabolic syndrome and diabetic kidney disease: a consistent dose–response association validated in an independent clinical cohort

**DOI:** 10.3389/fendo.2025.1724105

**Published:** 2026-01-22

**Authors:** Jie-Lin Qin, Jing Zhao, Ya-Li Chen, Xiao-Ke Li, Zhan-Zheng Zhao

**Affiliations:** 1 Department of General Medicine, The First Affiliated Hospital of Zhengzhou University, Zhengzhou, Henan, China; 2 School of Medicine, Zhengzhou University, Zhengzhou, Henan, China; 3 Nephrology Hospital, The First Affiliated Hospital of Zhengzhou University, Zhengzhou, Henan, China; 4 Department of Nephrology, The First Affiliated Hospital of Zhengzhou University, Zhengzhou, Henan, China

**Keywords:** diabetic kidney disease, HDL cholesterol, hyperglycemia, hypertension, metabolic syndrome, NHANES

## Abstract

**Background:**

Diabetic kidney disease (DKD) is a major complication of diabetes. The relative impact of individual metabolic syndrome (MetS) components and cumulative metabolic burden on DKD remains unclear.

**Methods:**

We analyzed 16,236 adults from NHANES 2011–2020. DKD was defined as reduced eGFR or increased urinary albumin-to-creatinine ratio in individuals with diabetes. MetS components included abdominal obesity, high blood pressure, high triglycerides, low high-density lipoprotein (HDL) cholesterol, and elevated glucose. Multivariable logistic regression evaluated associations of single components and MetS component count (MetS score) with prevalent DKD. Discrimination was assessed by receiver operating characteristic curves. External validation was performed in a retrospective cohort of 320 adults from The First Affiliated Hospital of Zhengzhou University.

**Results:**

In NHANES, 6.1% had DKD. Elevated glucose and low HDL cholesterol were independently associated with higher odds of DKD, whereas other components showed weaker or non-significant associations. DKD prevalence increased progressively with MetS score, and the score showed moderate discrimination (AUC 0.704). In the validation cohort (DKD prevalence 9.1%), elevated glucose, low HDL cholesterol, and high blood pressure were significant predictors of DKD. Each one-point increase in MetS score more than doubled the odds of DKD, with good discriminative performance (AUC 0.869; optimal cutoff ≥3 components).

**Conclusion:**

Elevated glucose and low HDL cholesterol are key MetS components associated with DKD, and a higher MetS component count confers progressively greater DKD risk. The MetS score may serve as a simple tool for DKD risk stratification in clinical practice.

## Introduction

Diabetic Kidney Disease (DKD) is one of the most serious and prevalent microvascular complications of diabetes mellitus, affecting up to 30–40% of patients with type 2 diabetes (1, 2). It is the leading cause of end-stage renal disease (ESRD) worldwide, imposing a substantial burden on patients, healthcare systems, and public health budgets (3, 4). DKD not only leads to progressive kidney failure but also markedly increases the risk of cardiovascular morbidity and mortality (5). Despite advances in glycemic control and renal-protective therapies, the incidence of DKD remains high, underscoring the need for improved strategies to identify high-risk individuals at earlier stages of the disease trajectory (6). Recognizing modifiable risk factors is therefore essential for implementing timely interventions to prevent or delay the onset of DKD.

Metabolic syndrome (MetS) is a cluster of interrelated cardiometabolic abnormalities—including central obesity, hypertension, dyslipidemia (high triglycerides and low HDL cholesterol), and hyperglycemia—that collectively increase the risk of type 2 diabetes and cardiovascular disease (7–9). Recent evidence has also suggested a potential link between MetS and chronic kidney disease (CKD), including DKD (10, 11). However, most studies to date have either focused on the general population or combined various types of kidney dysfunction, without specifically examining DKD as a distinct clinical endpoint. Moreover, it remains unclear which individual components of MetS confer the greatest risk for DKD, whether the accumulation of multiple components has a dose-dependent effect, or how interactions between components (e.g., hyperglycemia and hypertension) influence DKD risk.

Understanding the relative contribution and combined impact of MetS components on DKD could inform early risk stratification and targeted prevention strategies in diabetic populations. Yet, few large-scale, population-based studies have comprehensively evaluated these associations using standardized definitions and objective biomarkers.

Given these gaps in the literature, the objective of this study was to evaluate the association between MetS components and the risk of DKD using a large, nationally representative sample from the U.S. adult population. Specifically, we aimed to (1) assess the independent associations of individual MetS components with DKD, (2) evaluate the dose–response relationship between MetS component count and prevalent DKD, and (3) investigate the joint association of hyperglycemia and hypertension with prevalent DKD.

## Methods

### Study design and participants

This was a cross-sectional analysis based on data from the National Health and Nutrition Examination Survey (NHANES). We used publicly available data from five continuous NHANES cycles (2011–2012, 2013–2014, 2015–2016, 2017–2018, and 2019–2020). A total of 50,123 adults aged ≥18 years were initially considered. We excluded individuals who were pregnant (n = 835), had missing data on one or more metabolic syndrome components (n = 9,774), lacked measurements of urinary albumin or creatinine (n = 6,183), or had incomplete information on key covariates including age, sex, race/ethnicity, blood pressure, BMI, and fasting glucose (n = 17,095). After applying these criteria, 16,236 participants were retained for the final analysis.

**Figure 1:**
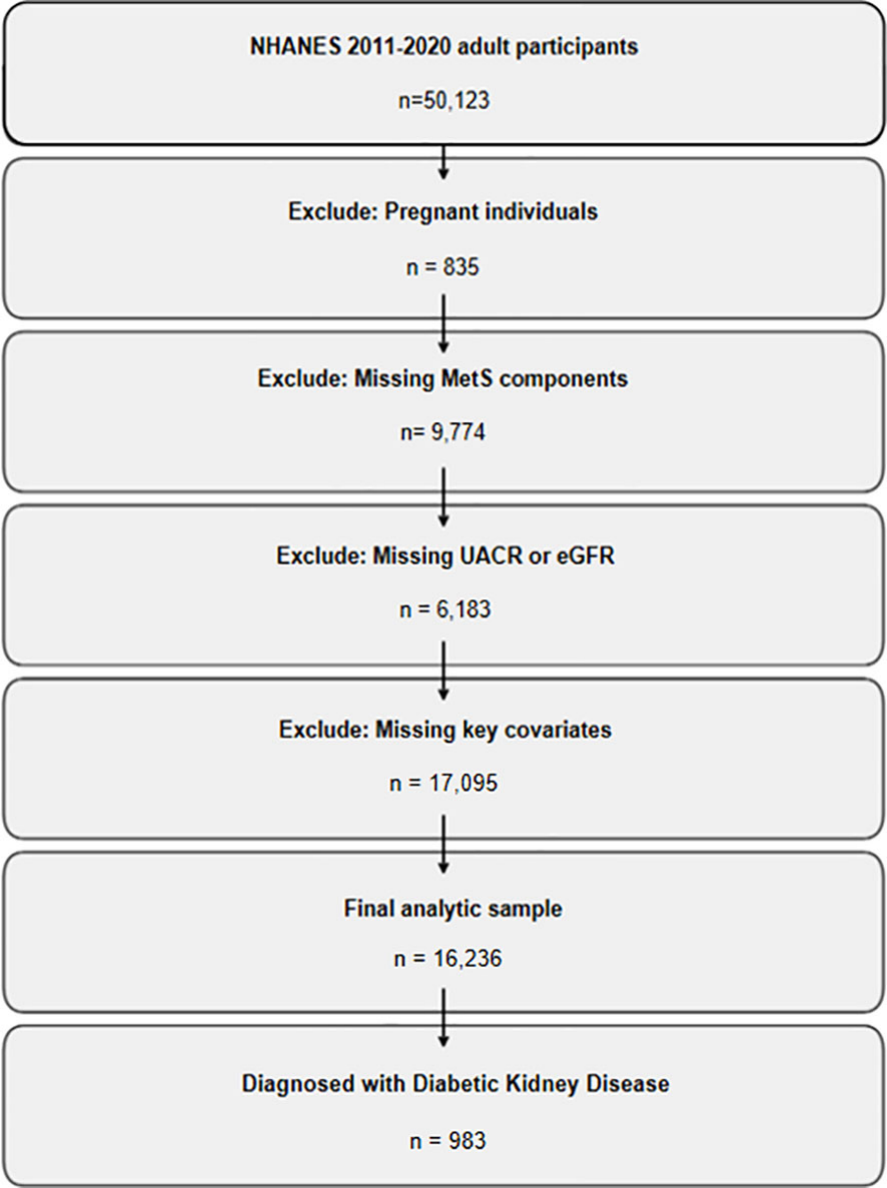
Flowchart of participant selection from NHANES 2011–2020.

DKD status was then determined within this analytic sample (see definition below). Participants who fulfilled the KDIGO-based criteria for DKD and had diabetes were classified into the DKD group. All remaining participants, including individuals with diabetes but without kidney involvement as well as those without diabetes, constituted the non-DKD comparison group. In total, 983 participants (6.1%) were classified as having DKD (**Figure 1**).

### Data collection and variables

All data were obtained from publicly available NHANES datasets across five cycles (2011–2020). We extracted information from multiple NHANES modules, including demographics, laboratory tests, physical examinations, and questionnaires.

### Outcome variable: diabetic kidney disease

DKD was defined according to the Kidney Disease: Improving Global Outcomes (KDIGO) 2020 guidelines, using estimated glomerular filtration rate (eGFR) and urinary albumin-to-creatinine ratio (UACR).

First, diabetes was identified if participants met any of the following criteria: (1) self-reported physician diagnosis of diabetes, (2) current use of antidiabetic medication or insulin, or (3) fasting plasma glucose ≥126 mg/dL (7.0 mmol/L).

Serum creatinine was used to calculate eGFR with the CKD-EPI equation, taking into account age, sex, and race. UACR was calculated by dividing urinary albumin concentration (URXUMA, mg/L) by urinary creatinine concentration (URXUCR, mg/dL) and converting the result to mg/g.

Among participants with diabetes, DKD was defined as the presence of either reduced kidney function (eGFR <60 mL/min/1.73 m²) or increased urinary albumin excretion (UACR ≥30 mg/g). Participants with diabetes who did not meet either criterion, together with all participants without diabetes, were categorized as not having DKD. Thus, DKD represents kidney damage or reduced kidney function occurring in the context of diabetes, whereas the reference group includes both diabetic and non-diabetic individuals without DKD.

### Main exposure: MetS components

Five components of MetS were assessed following the criteria of the National Cholesterol Education Program ATP III guidelines:

- Abdominal obesity: waist circumference ≥102 cm in men or ≥88 cm in women;
- High blood pressure: Systolic BP ≥ 130 mmHg, diastolic BP ≥ 85 mmHg, or antihypertensive medication use
- High triglycerides: Serum triglycerides ≥ 150 mg/dL
- Low HDL cholesterol: HDL < 40 mg/dL in men or < 50 mg/dL in women
- High glucose: Fasting glucose ≥ 100 mg/dL or use of antidiabetic medications
- A MetS score was calculated for each participant by summing the number of present components, ranging from 0 to 5.

### Covariates

The following covariates were included in the multivariable models:Age (continuous), Sex (male or female), Race/ethnicity (Non-Hispanic White, Non-Hispanic Black, Hispanic, Asian, or Other), Waist circumference and blood pressure were also described in descriptive statistics but not included in all models.

### Statistical analysis

All statistical analyses were conducted using R software (version 4.3.0). Descriptive statistics were used to summarize baseline characteristics, with continuous variables presented as means ± standard deviations and categorical variables as frequencies and percentages. Group comparisons between participants with and without DKD were performed using t-tests and chi-squared tests as appropriate. Logistic regression models were used to assess the associations between individual MetS components and DKD, adjusting for age, sex, and race/ethnicity. A dose–response relationship was evaluated by modeling the MetS score (range: 0–5) as a categorical predictor of DKD risk. Interaction between high blood pressure and high glucose was assessed by creating a four-level categorical variable and comparing odds ratios using multivariable logistic regression. Discriminative ability of the MetS score for predicting DKD was evaluated using receiver operating characteristic (ROC) curve analysis, and the area under the curve (AUC) was calculated. Two-sided *p*-values < 0.05 were considered statistically significant. To evaluate whether there was a graded association between the number of MetS components and prevalent DKD, we additionally modelled the MetS component count as a continuous variable and tested for a linear trend across categories (p for trend). As a sensitivity analysis, we restricted the NHANES sample to participants with diabetes and repeated the multivariable logistic regression models assessing the association between MetS component count and DKD.

### Ethical considerations

The study was based on secondary analysis of publicly available data from the NHANES, which is conducted by the National Center for Health Statistics (NCHS). All NHANES protocols were approved by the National Center for Health Statistics Research Ethics Review Board, and written informed consent was obtained from all participants at the time of data collection. Since the data used in this study are de-identified and publicly accessible, no additional ethical approval was required for the current analysis.

### External validation cohort

To externally validate the main findings, an independent retrospective cohort was collected from The First Affiliated Hospital of Zhengzhou University (Zhengzhou, China). A total of 320 adult patients with available biochemical and clinical data were included. Variable definitions and diagnostic criteria—including MetS components and DKD definition—were consistent with those used in the NHANES-based analysis. Because this validation study was retrospective and utilized de-identified data obtained from the hospital’s electronic medical records, formal ethical approval and individual informed consent were waived according to institutional review board policies.

## Results

### Baseline characteristics of participants

A total of 16,236 participants were included in the final analysis, among whom 983 (6.1%) were classified as having DKD. The baseline characteristics of participants stratified by DKD status are presented in **Table 1**.

**Table 1:** Baseline characteristics of participants.

Characteristics	Diabetic kidney disease		*P* value
	No (15253)	Yes (983)	
Age, years (mean (SD))	44.52 (17.00)	72.60 (11.13)	<0.001
Sex (%)			<0.001
Female	6213 (40.7)	205 (21.2)	
Male	9040 (59.3)	778 (78.8)	
Race (%)			<0.001
Asian	1197 (7.8)	71 (7.2)	
Black	3453 (22.6)	234 (23.8)	
Hispanic	3605 (23.6)	230 (23.4)	
Other	620 (4.1)	34 (3.5)	
White	6378 (41.8)	414 (42.1)	
Education (%)			<0.001
< High school	3139 (20.6)	174 (17.7)	
High school	4380 (28.7)	322 (32.8)	
Some college	4702 (30.8)	290 (29.5)	
College graduate	3032 (19.9)	197 (20.0)	
Income_ratio (mean (SD))	2.51 (1.20)	2.55 (1.24)	0.281
Smoker (%)			<0.001
No	11433 (75.0)	343 (35.6)	
Yes	3820 (25.0)	640 (64.4)	
Hba1c (mean (SD))	5.38 (1.01)	6.40 (1.37)	<0.001
Fasting_glu, mmol/L (mean (SD))	100.20 (35.03)	128.39 (64.37)	<0.001
BMI(mean (SD))	29.06 (6.92)	28.59 (6.95)	<0.001
Waist (mean (SD))	98.06 (16.52)	99.35 (17.12)	<0.001
SBP (mean (SD))	123.84 (18.21)	123.90 (18.30)	<0.001
DBP (mean (SD))	67.78 (12.93)	68.19 (13.15)	<0.001
Obesity (%)			<0.001
No	9597 (62.9)	620 (63.1)	
Yes	5656 (37.1)	363 (36.9)	
Abdominal_obesity (%)			<0.001
No	7287 (47.8)	464 (47.2)	
Yes	7966 (52.2)	519 (52.8)	
High_bp (%)			0.004
No	462 (3.0)	34 (3.5)	
Yes	14791 (97.0)	949 (96.5)	
High_trig (%)			<0.001
No	12283 (80.5)	802 (81.6)	
Yes	2970 (19.5)	181 (18.4)	
Low_hdl (%)			<0.001
No	10372 (68.0)	668 (68.0)	
Yes	4881 (32.0)	315 (32.0)	
High_glu (%)			<0.001
No	4729 (31.0)	0 (0)	
Yes	10524 (69.0)	983 (100)	
Mets_score (mean (SD))	1.60 (1.20)	1.70 (1.25)	<0.001

Compared with participants without DKD, those with DKD were markedly older (72.60 *vs*. 44.52 years, p < 0.001), more likely to be male (78.8% *vs*. 59.3%, p < 0.001), and had a slightly higher proportion of non-Hispanic White individuals (42.1% *vs*. 41.8%, p < 0.001). DKD participants also had higher body mass index (BMI), waist circumference, systolic blood pressure, and HbA1c levels than those without DKD (all *p* < 0.001).

Furthermore, the prevalence of individual MetS components, including obesity, high blood pressure, high triglycerides, low HDL cholesterol, and elevated glucose, was significantly higher in the DKD group (*p* < 0.05 for all). The mean MetS component count was slightly higher in participants with DKD than in those without DKD (1.70 *vs*. 1.60, p < 0.001), indicating a greater overall metabolic burden.

### Associations between individual MetS components and diabetic kidney disease

**Figure 2:**
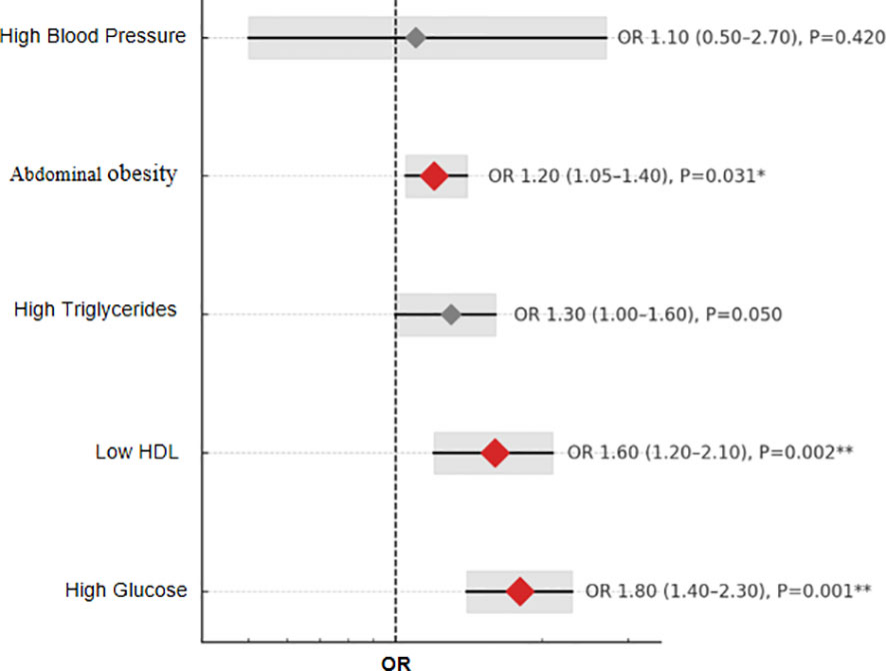
Adjusted OR for individual metabolic syndrome components associated with DKD.

The associations between individual MetS components and the risk of DKD were assessed using logistic regression models, and the results are visualized in **Figure 2**. In multivariable logistic regression models adjusted for age, sex, and race/ethnicity, several individual components of metabolic syndrome were significantly associated with increased risk of DKD. Compared with participants without the respective condition, those with abdominal obesity had a higher odds of DKD (OR = 1.20, 95% CI: 1.05–1.40, *p* = 0.031), and those with low HDL cholesterol had an even higher risk (OR = 1.60, 95% CI: 1.20–2.10, *p* = 0.002). Elevated glucose level was the strongest risk factor, showing a significant association with DKD (OR = 1.80, 95% CI: 1.40–2.30, *p* = 0.001). In contrast, high blood pressure (OR = 1.10, 95% CI: 0.50–2.70, *p* = 0.42) and high triglycerides (OR = 1.30, 95% CI: 1.00–1.60, *p* = 0.05) were not significantly associated with DKD after adjustment. These findings suggest that among MetS components, high glucose and low HDL-C are the strongest independent predictors of DKD.

### Dose–response relationship between MetS score and DKD risk

**Figure 3:**
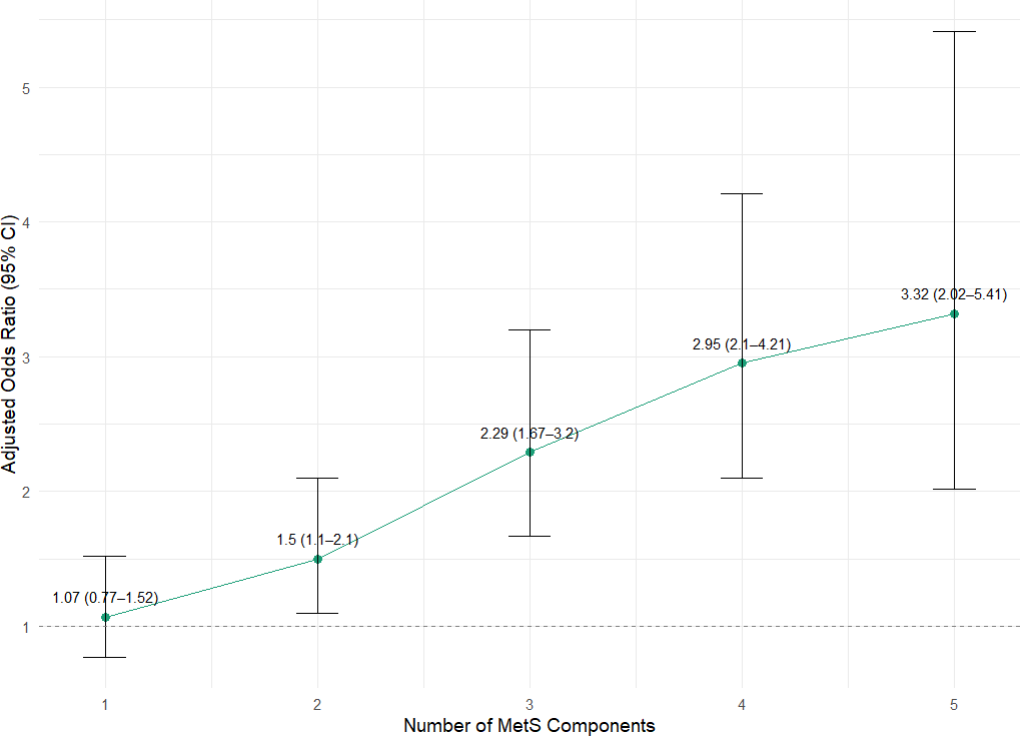
Dose–response relationship between MetS component count and the risk of DKD.

To further investigate the cumulative impact of metabolic dysfunction, we examined the association between the number of MetS components (MetS score, ranging from 0 to 5) and the risk of DKD. The results are illustrated in **Figure 3**.

Using multivariable logistic regression adjusted for age, sex, and race, we observed a clear dose–response relationship: as the MetS score increased, the odds of DKD rose progressively. Compared with individuals with a MetS score of 0 (reference group), the adjusted ORs for DKD were: A clear dose-response relationship was observed between the number of MetS components and the risk of DKD. Compared with individuals with a MetS score of 0, the adjusted odds ratios for DKD were 1.07 (95% CI: 0.77–1.52) for a score of 1, 1.50 (95% CI: 1.1–2.1) for a score of 2, 2.29 (95% CI: 1.67–3.2) for a score of 3, 2.95 (95% CI: 2.1–4.21) for a score of 4, and 3.32 (95% CI: 2.05–5.41) for a score of 5. This graded association suggests that a higher metabolic burden is strongly associated with elevated DKD risk, supporting a cumulative biological effect of multiple MetS components.

### Joint effect of high blood pressure and hyperglycemia on DKD risk

We further evaluated the combined effect of high BP and Glu on the risk of DKD using stratified logistic regression analysis. Participants were categorized into four groups based on the presence or absence of these two MetS components: (1) Neither condition, (2) Only high BP, (3) Only high Glu, (4) Both high BP and high Glu.

**Figure 4:**
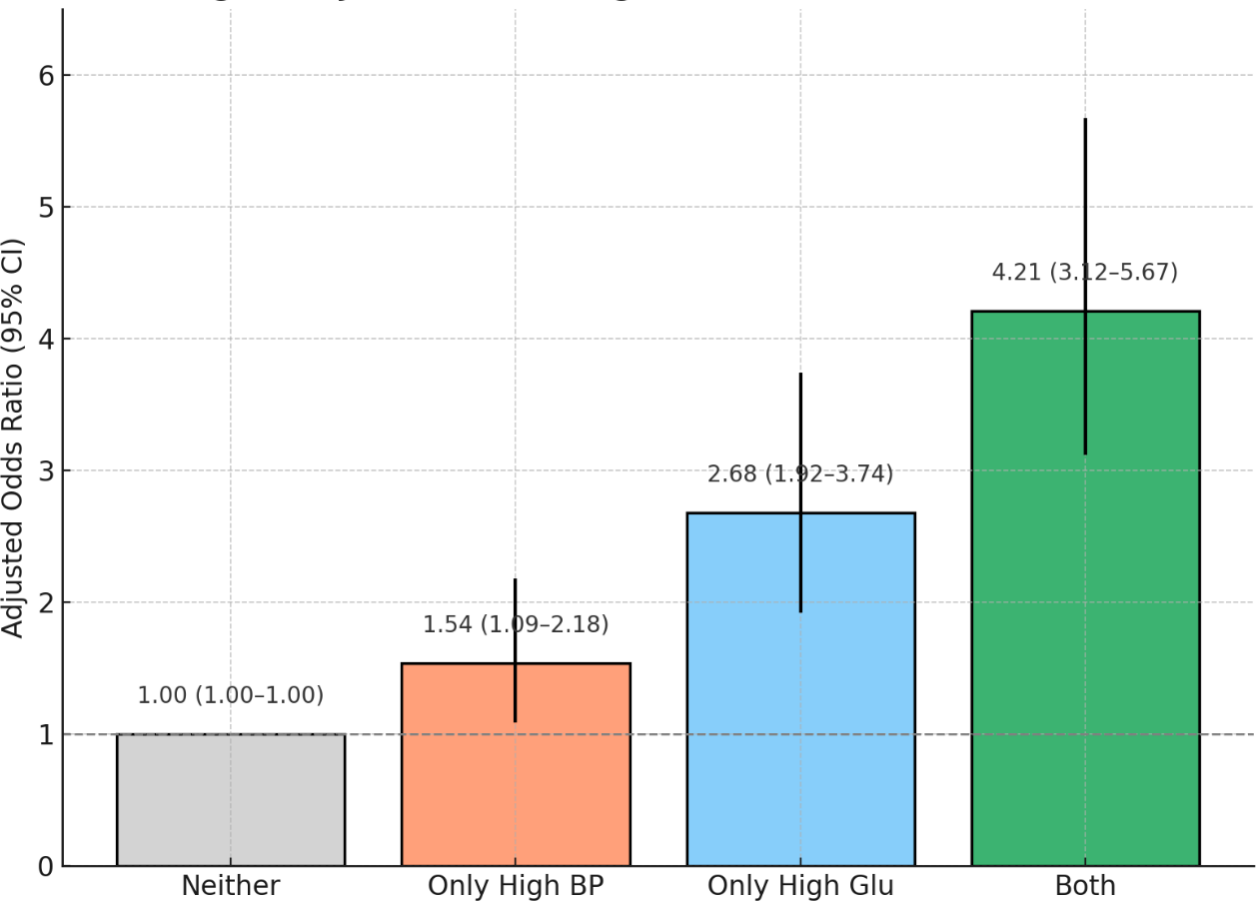
Joint effect of high blood pressure and high glucose on the risk of DKD.

Using multivariable logistic regression models adjusted for age, sex, and race/ethnicity, we observed that, compared with individuals with neither condition, the risk of DKD was significantly increased among those with only high BP (OR: 1.54, 95% CI: 1.09–2.18), only high glucose (OR: 2.68, 95% CI: 1.92–3.74), and both high BP and high glucose (OR: 4.21, 95% CI: 3.12–5.67). This dose-dependent elevation in risk indicates a potential synergistic interaction between these two metabolic factors. The reference group was individuals with neither condition. Results are shown in **Figure 4**. These findings indicate a synergistic effect between elevated blood pressure and hyperglycemia, where their coexistence substantially amplifies DKD risk beyond the sum of their individual effects.

### Discriminative ability of MetS score for identifying DKD

**Figure 5:**
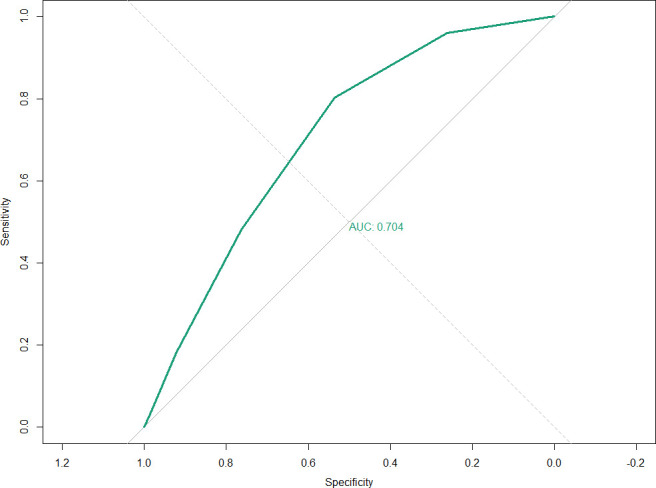
ROC curve for MetS score in discriminating DKD.

To evaluate the predictive performance of the MetS score for DKD, a ROC curve analysis was conducted. The area under the curve (AUC) was 0.704 (95% CI: 0.684–0.724), indicating acceptable discriminatory ability of the MetS score in identifying individuals with DKD. The ROC curve is shown in **Figure 5**. This result suggests that the MetS component count can serve as a useful risk stratification tool for DKD in population-based screening.

### External validation

To confirm the robustness and generalizability of the main findings, an independent external validation was conducted using a retrospective cohort from The First Affiliated Hospital of Zhengzhou University (Zhengzhou, China), which included 320 adult participants with complete biochemical and clinical information. Among them, 29 individuals (9.1%) fulfilled the diagnostic criteria for DKD.

Consistent with the NHANES-based results, hyperglycemia and low HDL cholesterol remained significant factors independently associated with DKD in the validation cohort. In multivariable logistic regression models adjusted for age, sex, and race, high glucose (OR = 5.74, 95% CI: 3.79–9.35), low HDL cholesterol (OR = 2.86, 95% CI: 1.36–5.76), and high blood pressure (OR = 2.86, 95% CI: 1.62–5.35) were significantly associated with an increased risk of DKD, whereas abdominal obesity (OR = 1.01, 95% CI: 0.53–1.69) and high triglycerides (OR = 0.97, 95% CI: 0.51–1.78) were not significant after adjustment.

A clear dose–response relationship was observed between MetS score and DKD risk, with each one-point increment in MetS score conferring a 2.23-fold higher odds of DKD (95% CI: 1.83–2.73). Furthermore, the joint-effect analysis demonstrated a pronounced synergistic interaction between elevated blood pressure and hyperglycemia: compared with participants with neither condition, those with both high BP and high glucose exhibited an approximately 11-fold greater risk of DKD (OR = 11.14, 95% CI: 6.73–17.27).

ROC analysis showed good discriminative performance of the MetS score in identifying prevalent DKD (AUC = 0.869), with an optimal cutoff of 3 points (sensitivity = 0.897, specificity = 0.722). These findings collectively validated the primary results derived from the NHANES population and confirmed the reproducibility of the associations between MetS burden and DKD in an independent clinical sample. The main results were further replicated in an independent clinical cohort (**Supplementary Figures S1**-**S3**; **Supplementary Tables S1**, **S2**).

### Integrated analysis of trend and sensitivity

When the MetS component count was modeled as a continuous variable, each one-component increase was associated with 1.42-fold higher odds of prevalent DKD (95% CI 1.32–1.54, p < 0.001) in the overall NHANES sample, and a significant linear trend across MetS categories was observed (p for trend < 0.001). This graded association remained robust in a sensitivity analysis restricted to participants with diabetes, where each additional MetS component was associated with 1.36-fold higher odds of DKD (95% CI 1.22–1.52, p < 0.001; p for trend < 0.001). In the external validation cohort, the MetS score demonstrated an even stronger graded association, with each one-component increase linked to 2.18-fold higher odds of DKD (95% CI 1.72–2.89, p < 0.001)and a consistently significant trend (**Supplementary Table S3**). Together, these findings support a stable and reproducible relationship between increasing metabolic burden and DKD across populations.

## Discussion

In this nationally representative cross-sectional study based on NHANES data from 2011 to 2020, we found that approximately 6.1% of U.S. adults met the criteria for DKD. Our results demonstrated that among the five components of MetS, high glucose and low HDL cholesterol were the strongest independent risk factors for DKD. A clear dose–response relationship was observed between the MetS score and DKD risk, with individuals exhibiting higher MetS scores facing significantly elevated odds of DKD. Furthermore, a synergistic interaction between high blood pressure and high glucose was identified, suggesting that the coexistence of these two conditions substantially amplifies DKD risk. Finally, the MetS score demonstrated moderate discriminative ability (AUC = 0.704) for identifying prevalent DKD, indicating its potential utility in population-level risk stratification.

This study is, to our knowledge, among the first to comprehensively evaluate the relationship between MetS and DKD in a population-based setting, using standardized definitions and objective laboratory data across five continuous NHANES cycles. A key strength and novelty of this study is the cross-cohort validation of the graded association between metabolic burden and DKD. Demonstrating consistent findings in both a nationally representative population and an independent clinical cohort provides strong support for the robustness and generalizability of the MetS–DKD relationship.

Our findings are consistent with prior studies that have highlighted the strong association between hyperglycemia and DKD (6). Chronic hyperglycemia is a well-established contributor to glomerular hyperfiltration, mesangial expansion, and progressive renal damage, as described in both the UKPDS and ADVANCE trials (12–14). Similarly, the inverse relationship between HDL cholesterol levels and DKD risk observed in our analysis aligns with previous epidemiological evidence suggesting that low HDL-C is linked to increased inflammation, endothelial dysfunction, and oxidative stress—mechanisms that may accelerate renal injury in diabetic populations (15, 16). However, in contrast to some earlier reports that identified obesity and hypertriglyceridemia as strong predictors of DKD, these components were not independently significant in our fully adjusted models (17, 18). This discrepancy may be due to differences in population structure, definitions of metabolic syndrome, or the inclusion of glycemic control variables in our multivariable analysis, which may attenuate the effects of obesity-related markers (18). Notably, the observed additive effect of high blood pressure and hyperglycemia on DKD risk reinforces previous clinical evidence that co-management of hypertension and glycemic control is critical in preventing renal complications in patients with type 2 diabetes (19, 20).

The associations observed between individual MetS components and DKD are supported by well-established biological mechanisms. Hyperglycemia contributes to DKD pathogenesis through multiple pathways, including the accumulation of advanced glycation end products (AGEs), activation of the polyol pathway, oxidative stress, and chronic low-grade inflammation, all of which result in structural and functional damage to glomeruli and renal tubules (21–23). Persistent high glucose levels also promote mesangial expansion and basement membrane thickening, accelerating the decline in glomerular filtration (24).

Low HDL cholesterol, traditionally considered protective against atherosclerosis, has also been implicated in renal injury (25). HDL exerts anti-inflammatory, antioxidative, and endothelial-protective effects. A deficiency in HDL may impair cholesterol efflux, promote vascular inflammation, and exacerbate oxidative stress within the renal microvasculature, thereby increasing susceptibility to nephron damage (26). The observed synergistic effect of high blood pressure and hyperglycemia may be explained by their converging impact on glomerular hemodynamics. Hypertension increases intraglomerular pressure and mechanical stress, while hyperglycemia enhances sodium-glucose cotransporter-2 (SGLT2) activity and tubular workload, both contributing to glomerular hyperfiltration and endothelial dysfunction (27–29). The coexistence of these two factors creates a pro-injury renal environment that accelerates nephropathy progression.

Moreover, the accumulation of multiple MetS components may reflect an overarching state of metabolic inflammation and insulin resistance, which are increasingly recognized as central mechanisms in the development of both cardiovascular and renal complications in diabetes (7, 30).

The external validation performed in a retrospective clinical cohort from The First Affiliated Hospital of Zhengzhou University provided further support for the robustness of our findings. The direction and magnitude of associations observed in this independent sample closely mirrored those derived from the NHANES dataset. Specifically, hyperglycemia, low HDL cholesterol, and high blood pressure remained the predominant risk factors for DKD, whereas triglycerides and abdominal obesity showed weaker and nonsignificant associations after multivariable adjustment. These results reinforce the central role of glycemic and lipid dysregulation, together with elevated blood pressure, in the pathophysiological cascade leading to diabetic kidney injury. Because NHANES does not provide definitive diabetes type, we were unable to analyze type 1 and type 2 diabetes separately. However, type 2 diabetes accounts for the overwhelming majority in adults, and therefore the potential misclassification is unlikely to substantially influence the observed associations.

Importantly, the validation cohort reproduced the dose–response relationship between cumulative metabolic burden and DKD risk, with each additional MetS component conferring over a twofold increase in odds of DKD. The synergistic effect of coexisting hyperglycemia and hypertension was also reaffirmed, underscoring that simultaneous metabolic and hemodynamic stress markedly amplifies renal vulnerability. The discriminative performance of the MetS score was even stronger in the validation sample (AUC = 0.869), suggesting that the score may serve as a practical, easily obtainable index for DKD risk stratification in clinical settings.

This study has several notable strengths. First, it utilized data from NHANES, a large, nationally representative survey employing standardized protocols and rigorous quality control, which enhances the generalizability of our findings to the U.S. adult population. Second, we applied objective and widely accepted definitions of MetS and DKD, incorporating laboratory measures such as fasting glucose, serum creatinine, and UACR. Third, the study included a comprehensive analytic framework, incorporating multivariable adjustment, dose–response modeling, interaction analysis, and ROC-based discrimination assessment, which provides robust insight into the relationship between metabolic burden and DKD risk.

However, several limitations should be acknowledged. First, the cross-sectional design precludes causal inference, and the relationships identified should be interpreted as associations with prevalent DKD rather than causal determinants. Longitudinal cohort studies are needed to clarify temporal sequence and confirm causality. Second, DKD was defined using single-time-point laboratory values, rather than persistent measurements over time, which may overestimate or misclassify chronic kidney disease. Third, although we adjusted for major confounders such as age, sex, and race/ethnicity, residual confounding from unmeasured variables (e.g., diet, physical activity, medication use) cannot be fully excluded. Lastly, the external validation cohort was derived from a single hospital and had a relatively small sample size, which may limit representativeness.

## Conclusion

Our findings from both a nationally representative cohort and an independent hospital-based sample consistently demonstrate that the metabolic syndrome, particularly hyperglycemia, low HDL cholesterol, and hypertension, is strongly and consistently associated with the presence of DKD. The observed dose–response pattern and successful external validation underscore the robustness and generalizability of these associations. The MetS score exhibits strong discriminative performance and may serve as a promising clinical indicator for early DKD risk stratification. These results provide an evidence-based foundation for further interventional studies focusing on metabolic optimization to prevent DKD progression.

## Data Availability

The original contributions presented in the study are included in the article/**Supplementary Material**. Further inquiries can be directed to the corresponding author.
