# Supplementary material for: Metabolic syndrome and diabetic kidney disease: a consistent dose–response association validated in an independent clinical cohort
Source: Front Endocrinol (Lausanne). 2026 Jan 22;16:1724105. doi: 10.3389/fendo.2025.1724105 (PMC12872542; doi:10.3389/fendo.2025.1724105)
Supplement: Supplementary file 1 [file DataSheet1.doc]

| **Table S1. Baseline characteristics of the validation cohort (n = 320) stratified by DKD status** | | | | |
| --- | --- | --- | --- | --- |
| **Variable** | **No_DKD(Mean±SD)** | **DKD(Mean±SD)** | **Overall(Mean±SD)** | **N** |
| Age (year) | 48.36 ± 16.14 | 59.81 ± 16.49 | 49.40 ± 16.47 | 320 |
| BMI | 28.46 ± 4.22 | 32.92 ± 4.03 | 28.86 ± 4.39 | 320 |
| Waist | 97.35 ± 9.92 | 105.73 ± 9.01 | 98.11 ± 10.12 | 320 |
| SBP | 121.42 ± 14.57 | 139.83 ± 16.10 | 123.08 ± 15.61 | 320 |
| DBP | 67.79 ± 10.24 | 72.55 ± 9.38 | 68.22 ± 10.24 | 320 |
| Triglycerides | 127.47 ± 72.68 | 184.48 ± 107.51 | 132.63 ± 78.01 | 320 |
| HDL | 57.88 ± 9.66 | 49.94 ± 10.24 | 57.16 ± 9.96 | 320 |
| Fasting_glucose (mmol/L) | 91.08 ± 20.07 | 140.65 ± 12.79 | 95.57 ± 24.16 | 320 |
| eGFR (mL/min/1.73 m²) | 96.91 ± 19.30 | 73.46 ± 17.69 | 94.79 ± 20.29 | 320 |
| UACR (mg/g) | 25.09 ± 33.48 | 99.54 ± 104.92 | 31.84 ± 49.43 | 320 |
| Sex |  |  |  |  |
| Female | 152 | 16 | 168 | 168 |
| Male | 139 | 13 | 152 | 152 |
| Abdominal obesity |  |  |  |  |
| False | 121 | 2 | 123 | 123 |
| True | 170 | 27 | 197 | 197 |
| High BP |  |  |  |  |
| False | 197 | 5 | 202 | 202 |
| True | 94 | 24 | 118 | 118 |
| High TG |  |  |  |  |
| False | 207 | 15 | 222 | 222 |
| True | 84 | 14 | 98 | 98 |
| Low HDL |  |  |  |  |
| False | 265 | 17 | 282 | 282 |
| True | 26 | 12 | 38 | 38 |
| High Glucose |  |  |  |  |
| False | 188 | 0 | 188 | 188 |
| True | 103 | 29 | 132 | 132 |

| **Table S2. Adjusted odds ratios of individual MetS components for DKD** | | | | | |
| --- | --- | --- | --- | --- | --- |
| **Term** | **coef** | **OR** | **CI_low** | **CI_high** | **label** |
| Abdominal obesity | 0.009 | 1.01 | 0.534 | 1.692 | Abdominal Obesity |
| High BP | 1.052 | 2.87 | 1.621 | 5.351 | High BP |
| High Trig | -0.033 | 0.97 | 0.508 | 1.782 | High Trig |
| Low Hdl | 1.051 | 2.86 | 1.359 | 5.764 | Low Hdl |
| High Glu | 1.747 | 5.74 | 3.785 | 9.349 | High Glu |


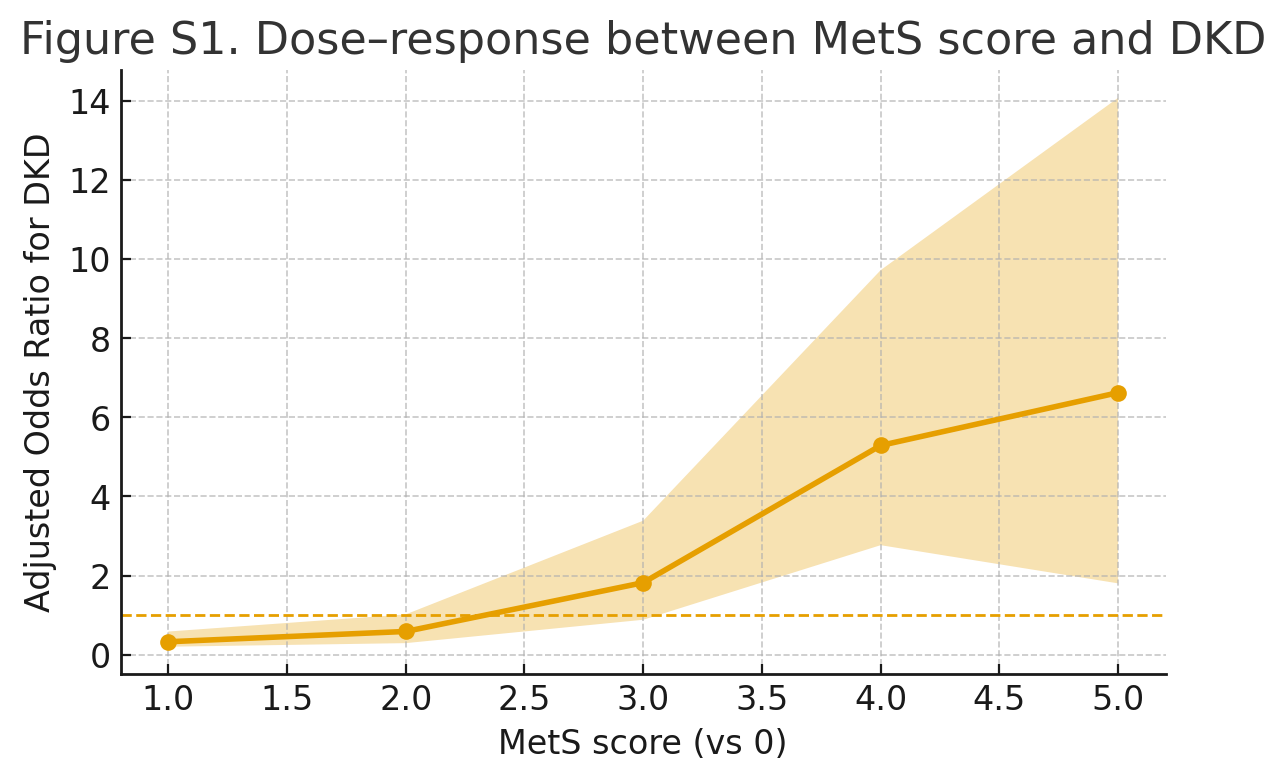


**Figure S1.** Dose–response relationship between MetS score and DKD.


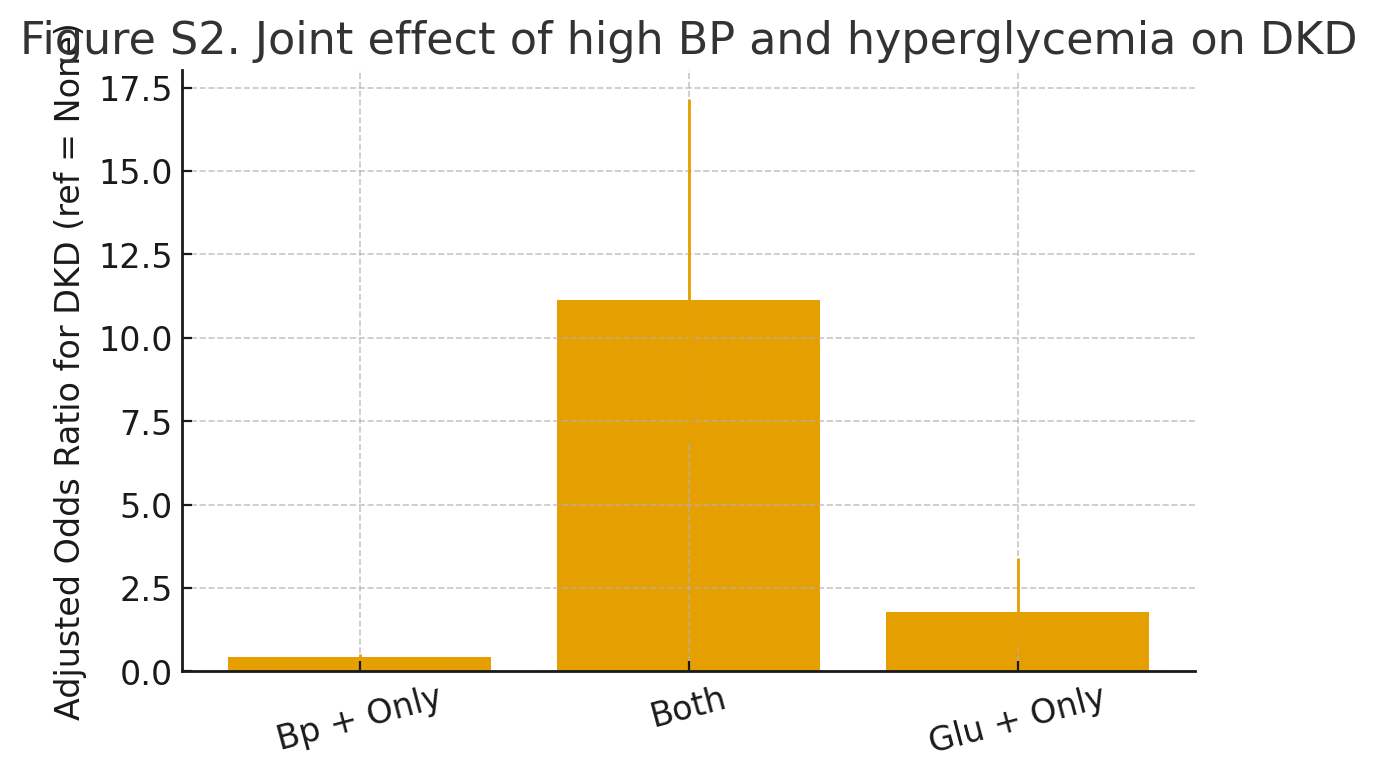


**Figure S2.** Joint effect of high blood pressure and hyperglycemia on the risk of DKD.


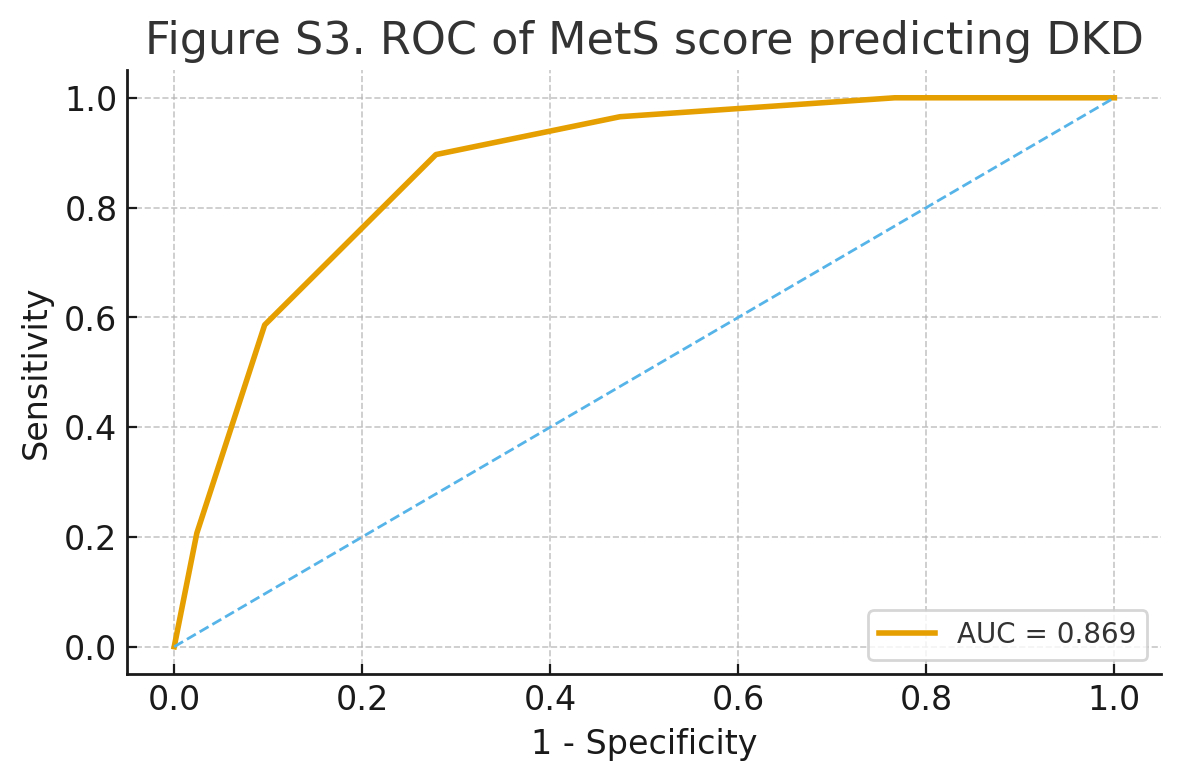


**Figure S3.** ROC curve of the MetS score for predicting DKD.

| **Table S3. Association between MetS component count and prevalent DKD: main and sensitivity analyses.** | | | | | |
| --- | --- | --- | --- | --- | --- |
| **Population / Model** | **n** | **DKD cases, n (%)** | **OR per 1-component increase (95% CI)** | **p-value** | **p for trend** |
| NHANES  (full sample) | 16,236 | 983 (6.1) | 1.42 (1.32–1.54) | <0.001 | <0.001 |
| NHANES (diabetes only) | 3,480 | 610 | 1.36 (1.22–1.52) | <0.001 | <0.001 |
| External validation cohort | 320 | 29 (9.1) | 2.18 (1.72–2.89) | <0.001 | <0.001 |
